# Characterization of bone metabolism in hungarian psoriatic arthritis patients: a case–control study

**DOI:** 10.1186/s12891-021-03952-z

**Published:** 2021-01-12

**Authors:** Zsófia Pethő, Edit Kalina, Zoltán Pap, Katalin Hodosi, Rebeka Falcsik, Ádám Balogh, Zoltán Szekanecz, Harjit Pal Bhattoa

**Affiliations:** 1 Kalman Laki Doctoral School of the University of Debrecen, Division of Rheumatology, Department of Internal Medicine, Faculty of Medicine, University of Debrecen, Debrecen, Hungary; 2 Department of Laboratory Medicine, Faculty of Medicine, University of Debrecen, Nagyerdei Blvd. 98, Debrecen, H-4032 Hungary; 3 Kalman Laki Doctoral School of the University of Debrecen, Department of Traumatology and Hand Surgery, Faculty of Medicine, University of Debrecen, Debrecen, Hungary; 4 Division of Rheumatology, Department of Internal Medicine, Faculty of Medicine, University of Debrecen, Debrecen, Hungary; 5 Regional Osteoporosis Center, Department of Obstetrics and Gynecology, Faculty of Medicine, University of Debrecen, Debrecen, Hungary

**Keywords:** Psoriatic arthritis, Areal and volumetric BMD, FRAX, Bone metabolism, Disease activity

## Abstract

**Background:**

Skeletal manifestations are predominant in psoriatic arthritis (PsA). The aim of this cross-sectional, case-control study is the complex assessment of areal and volumetric bone mineral density (BMD), fracture risk, vitamin D status and bone turnover markers, and its association with disease-related variables.

**Methods:**

Lumbar spine (L1-L4) and femoral neck (FN) areal, and distal radius (DR) volumetric BMD, 10-year probability of major and hip osteoporotic fracture as assessed by the fracture risk assessment (FRAX) tool, markers of bone metabolism and disease activity were assessed.

**Results:**

Upon comparison of the disease and age- and sex-matched control groups, there was a statistically significant difference in FN areal (0.952 (0.607–1.292) g/cm^2^ vs. 1.016 (0.760–1.550) g/cm^2^; *p* = 0.001) and DR total volumetric (284.3 (138.9–470.3) mg/cm^3^ vs. 367.0 (287.0–412.0) mg/cm^3^; *p* < 0.001) BMD, 10 year probability for major osteoporotic (3.7% (0.7–32%) vs. 2.6% (0–17.5%); *p* = 0.003) and hip (0.4% (0–16%) vs. 0.05% (0–6.1%); *p* = 0.002) fracture and 25-hydroxyvitamin D status (47.5 (10–120) nmol/L vs. 64 (10–137; *p* < 0.001) nmol/L). As compared to areal assessment, volumetric BMD measurements identified a significantly higher number of patients with low bone mineral density (T-Score ≤ − 1.00) (34% vs. 88%, *p* < 0.001). Upon multiple linear regression analysis, disease activity score, as determined by DAS28 assessment, was an independent predictor of 10-year probability for major osteoporotic fracture (*B* (95%CI) = 1.351 (0.379–2.323); *p* = 0.007).

**Conclusion:**

In the studied PsA cohort, disease activity was an independent predictor of 10-year probability for a major osteoporotic fracture, and complemented assessment of volumetric and areal BMD assured better efficacy at identifying those with low bone mineral density.

## Background

The prevalence of psoriasis is estimated at 1–3% of the world’s population [1]. It is a common skin disease associated with multiple comorbidities and the most prevalent coexisting condition, psoriatic arthritis (PsA) develops in 19.7% of psoriatic patients [2].

In majority of the patients, arthritis is manifested following psoriasis, and in others, it develops simultaneously or before the appearance of skin lesions [3]. Spinal manifestations resemble those in ankylosing spondylitis, and destructive peripheral joint characteristics resemble those of rheumatoid arthritis [4]. Pathologic de novo bone formation, including joint ankylosis, and syndesmophyte formation, characteristically localize to sites of soft-tissue inflammation surrounding the enthesis [5].

Osteoporosis is a systemic skeletal disease characterized by reduced bone mass, microarchitectural damage and increased fragility of bone [6]. Bone loss is a common comorbidity in chronic inflammatory diseases including PsA [7]. A systematic review by Chandran et al., where 21 studies conducted between 2001 and 2014 were included, highlighted the gap in our current knowledge given the discordant findings reported on the prevalence of low bone mineral density in PsA [8].

Osteoporosis has been operationally defined on the basis of bone mineral density (BMD) assessment. The most widely validated technique to measure BMD is dual energy X-ray absorptiometry (DXA), and diagnostic criteria based on the T-score for BMD are a recommended criteria for prescription of pharmaceutical interventions in osteoporosis [9]. According to the WHO criteria, osteoporosis is defined as a BMD that lies at least 2.5 SD below young healthy average, and therefore 2.5 or more SD below the average healthy young adult (T-score ≤ − 2.5 SD) [10, 11]. A major problem with BMD measurement is that these tests alone are not optimal for the detection of individuals at high risk of fracture [12].

On the other hand, peripheral quantitative computed tomography (pQCT) is excellent at three-dimensional quantification of cortical and trabecular bone at various regions of interest, *albeit*, is not recommended for conventional diagnostic classification [13]. Recent reports have discussed the techniques’ utility in patients suffering from inflammatory rheumatic disease [14, 15].

Fragility fractures are defined as fractures that occur spontaneously or following low-trauma and are potential cause of severe disability along with increased mortality risk [10]. Major advance in fragility fracture risk stratification has been achieved by the development of the fracture risk assessment tool (FRAX). The FRAX tool is based on country specific population-based cohorts that assimilate the risks associated with clinical risk factors (age, sex, body mass index, prior fragility fracture, parental history of hip fracture, steroid use, smoking, alcohol intake, disorders strongly associated with osteoporosis and rheumatoid arthritis) and BMD at the femoral neck [16]. The percentage output is a 10-year probability of hip fracture and the 10-year probability of a major osteoporotic fracture (clinical spine, forearm, hip, or shoulder fracture) [16]. Given the clinical, social and economic burden of osteoporotic fractures, the FRAX tool is considered ideal in identifying those at risk and advancing timely preventive or therapeutic interventions.

Presently, studies on complex assessment of areal and volumetric bone mineral density, fracture risk with the FRAX tool, vitamin D and markers of bone turnover in the same cohort of PsA patients are unavailable and the aim of the present cross-sectional, case-control study is to examine bone metabolism and evaluate its association with disease variables.

## Methods

### Patients and controls

We enrolled a total of 118 patients presenting for regular scheduled follow-up at the Division of Rheumatology, Faculty of Medicine, University of Debrecen, Hungary between September 2017 and June 2018. All were diagnosed with PsA as per the Classification Criteria for Psoriatic Arthritis (CASPAR) [17]. Data from patients with psoriatic arthritis was compared to age- and gender-matched volunteers. The control group was constituted by volunteers from our hospital staff or companions (friends or relatives) accompanying the PsA patients to their routine follow-up visit. All volunteers were briefed verbally about the aim of the study and the procedures involved, and all study participants gave written informed consent. For the control group, inclusion criteria were: generally known to be healthy, community dwelling and ambulatory; and exclusion criteria were known prevalent metabolic bone disease or rheumatological condition, any malignancy, liver or renal disease (routine laboratory results for alkaline phosphatase, gamma-glutamyl transpeptidase, alanine aminotransferase, aspartate aminotransferase, total bilirubin, lactate dehydrogenase, urea, creatinine, uric acid and cholinesterase twice the upper limit of normal resulted in exclusion). Volunteers with the closest dates of birth and blood drawing were selected for pairing with the PsA patients. All participants were Hungarian nationals as assessed by proof of identity. The study was performed according to the Declaration of Helsinki and approved by the Hungarian Scientific Research Council Ethical Committee (approval No. 14804–2/2011/EKU).

### Disease activity

All patients underwent physical examination and disease severity assessment. Disease Activity Score in 28 joints (DAS28), in those with peripheral involvement, Bath Ankylosing Spondylitis Disease Activity Index (BASDAI), in those with spinal involvement, and Psoriasis Area and Severity Index (PASI) were calculated [18–20].

### Laboratory

Blood sampling was done after overnight fasting to measure levels of osteocalcin (OC), C-terminal telopeptides of type-I collagen (CTx), procollagen type I amino-terminal propeptide (PINP), parathyroid hormone (PTH) and 25-hydroxyvitamin D (25OHD). Serum OC, CTx, PINP and PTH were measured using electrochemiluminescence immunoassay (Roche Diagnostics GmbH, Mannheim, Germany). The automated Liaison DiaSorin total 25OHD chemiluminescence immunoassay (CLIA) (DiaSorin Inc., Stillwater, MN, USA) was used to analyze serum 25OHD. The inter-assay CV was < 4% for OC (lower detection limit: 0.5 μg/L, upper detection limit: 300 μg/L), < 7% for CTx (lower detection limit: 0.010 μg/L, upper detection limit: 6 μg/L), < 6% for PINP (lower detection limit: 5 μg/L, upper detection limit: 1200 μg/L), < 7% for PTH (lower detection limit: 0.127 pmol/L, upper detection limit: 530 pmol/L) and < 7.8% for 25OHD (lower detection limit: 10 nmol/L, upper detection limit: 375 nmol/L). As suggested by Dawson-Hughes et al., hypovitaminosis D was defined as 25OHD levels < 75 nmol/l [21]. The erythrocyte sedimentation rate (ESR) was assessed with the Westergren method [22] and used in the calculation of the DAS28 score.

### Dual energy X-ray absorptiometry (DXA)

The LUNAR Prodigy (GE-Lunar Corp., Madison, WI, USA) densitometer was used to perform DXA examination to measure L1–L4 lumbar spine and left femoral neck (FN) areal BMD. Using the anatomical spine phantom measured daily, the coefficient of variation (CV) of the technique was 0.8%. Bone mineral density was expressed as a T-score, normalcy, low BMD and osteoporotic BMD were defined according to the WHO classification [11].

### Peripheral quantitative computer tomography (pQCT)

Single-slice pQCT assessments of the ultradistal region of the left forearm were performed using a Stratec XCT-2000 instrument (Stratec Medizintechnik GmbH, Pforzheim, Germany) as described by Juhasz et al. [14]. In summary, distal sites at 4% of the radius length mainly contain trabecular bone. Peripheral quantitative computer tomography can differentiate between cortical and trabecular bone. Total, trabecular, and cortical BMD values are expressed as mg/cm^3^. The applied setting to acquire the image was 0.59 mm voxel. Analysis was done with the XCT 6.00 B software (Stratec Medizintechnik GmbH, Pforzheim, Germany) with measuring mask set to radius and threshold density to 269 mg/cm^3^ to define trabecular bone.

### Fracture risk assessment tool (FRAX)

A trained study nurse administered a questionnaire to assess the country-specific FRAX index using the tool available online [16]. The data collected were age, sex, weight, height, non-traumatic fracture in the history, parental history of hip fracture, current smoking and alcohol consumption habits, corticosteroid use, diagnosis of rheumatoid arthritis or any condition known to cause low bone mass and femoral BMD.

### Statistical analysis

Descriptive statistics are presented as median and range. The Kolmogorov-Smirnov test was used to check for normality of distribution. The Wilcoxon signed ranks test was used to compare the age- and gender-matched pairs. The Spearman’s ρ was calculated for correlation analysis. Univariate and multiple regression analysis using the stepwise method was used to determine correlations and independent associations between parameters. Dual energy X-ray absorptiometry, pQCT and FRAX parameters were the dependent variables and other parameters were independent variables. The β standardized linear coefficients showing linear correlations between two parameters were determined. The *B* (95%CI) regression coefficient indicated independent association between the dependent and independent variable during changes. *p* values < 0.05 indicated statistical significance. All analyses were performed using the SPSS Statistics software, version 25.0 (IBM Corps., Armonk, NY, USA).

## Results

Patients (*n* = 118) presenting with psoriatic arthritis, confirming to the CASPAR diagnostic criteria, were included in this cross-sectional, analyst blinded, age- and sex-matched, case-control study [17]. The median age (range) of the patients was 54 (25–85) years, with a women:men ratio of 67:51. The median (range) disease duration for psoriasis and arthritis was 16 (1–72) and 9 (0–39) years, respectively. In a small percentage of the patients (*n* = 14, 12%), the diagnosis of psoriasis was confirmed following the diagnosis of arthritis (on an average within 5 years). Compared to the controls, L1-L4 areal BMD (1.274 (0.920–1.760) gm/cm^2^ vs. 1.170 (0.793–1.184) gm/cm^2^; *p* < 0.001), FN areal BMD (1.016 (0.760–1.550) gm/cm^2^ vs. 0.952 (0.607–1.292) gm/cm^2^; *p* = 0.001), distal radius (DR) total volumetric BMD (367.0 (287.0–412.0) gm/cm^3^ vs. 284.3 (138.9–470.3) gm/cm^3^; *p* < 0.001), DR trabecular volumetric BMD (207.0 (145.0–255.0) gm/cm^3^ vs. 190.0 (63.0–351.5) gm/cm^3^; *p* = 0.002), DR cortical volumetric BMD (537.0 (411.0–537.0) gm/cm^3^ vs. 365.9 (175.8–686.5) gm/cm^3^; *p* < 0.001) and 25OHD (64 (10–137) nmol/L vs. 47.5 (10–120) nmol/L; *p* < 0.001) was significantly lower, and the 10 year probability for a major osteoporotic fracture (2.6 (0–17.5) % vs. 3.7 (0.7–32) %; *p* = 0.003) and hip fracture (0.05 (0–6.1) % vs. 0.4 (0–16) %; *p* = 0.002), CTx (0.200 (0.100–0.511) μg/L vs. 0.255 (0.040–1.090) μg/L; *p* < 0.001) and PINP (35.0 (8.2–72.5) μg/L vs. 46.2 (11.0–253.7) μg/L; *p* < 0.001) were significantly higher in the PsA group (Table 1). The frequency of normal, low and osteoporotic BMD in PsA patients at different regions of interest is presented in Table 2. Areal and volumetric BMD showed statistically significant correlation with each other (Table 3).

**Table 1:** Subject characteristics

Parameters	All patients (***n*** = 118)	All controls (***n*** = 118)	***p*** value
Age, years (median, range)	54 (25–85)	54 (25–85)	1.000
Women:Men	67:51	67:51	1.000
DAS28, % (median, range) (*n* = 110)	2.37 (0.49–5.85)	–	–
BASDAI, % (median, range) (*n* = 8)	1.42 (0.02–3.08)	–	–
PASI, % (median, range)	1.65 (0–29.40)	–	–
Arthritis duration, years (median, range)	9 (0–39)	–	–
Psoriasis duration, years (median, range)	16 (1–72)	–	–
FRAX Major, % (median, range) (*n* = 100)	3.7 (0.7–32)	2.6 (0–17.5)	0.003
FRAX Hip, % (median, range) (*n* = 100)	0.4 (0–16)	0.05 (0–6.1)	0.002
10 year probability of major osteoporotic fracture ≥20% (n, %)	1 (1%)	0 (0%)	–
10 year probability of hip fracture ≥3% (n, %)	8 (8%)	4 (4%)	–
L1-L4 areal BMD, g/cm^2^ (median, range)	1.170 (0.793–1.184)	1.274 (0.920–1.760)	< 0.001
Femoral neck areal BMD, g/cm^2^ (median, range)	0.952 (0.607–1.292) (*n* = 117*)	1.016 (0.760–1.550)	0.001
Distal radius total volumetric BMD, mg/cm^3^ (median, range)	284.3 (138.9–470.3)	367.0 (287.0–412.0)	< 0.001
Distal radius trabecular volumetric BMD, mg/cm^3^ (median, range)	190.0 (63.0–351.5)	207.0 (145.0–255.0)	0.002
Distal radius cortical volumetric BMD, mg/cm^3^ (median, range)	365.9 (175.8–686.5)	537.0 (411.0–537.0)	< 0.001
Calcium, mmol/L (median, range)	2.4 (2.2–2.7)	2.3 (2.1–2.7)	< 0.001
Phosphate, mmol/L (median, range)	0.96 (0.6–1.6)	1.10 (0.6–1.51)	< 0.001
Osteocalcin, μg/L (median, range)	17.7 (5–77)	17.9 (8.6–33)	0.186
CTx, μg/L (median, range)	0.255 (0.040–1.090)	0.200 (0.100–0.510)	< 0.001
PINP, μg/L (median, range)	46.2 (11.0–253.7)	35.0 (8.2–72.5)	< 0.001
PTH, pmol/L (median, range)	4.52 (1.43–11.69)	3.78 (1.6–9.6)	< 0.001
25OHD, nmol/L (median, range)	47.5 (10–120)	64 (10–137)	< 0.001
25OHD < 75 nmol/L	79% (*n* = 93)	58% (*n* = 68)	–
25OHD < 50 nmol/L	49% (*n* = 58)	28% (*n* = 33)	–

*DAS28* Disease activity Score in 28 joints, *BASDAI* Ankylosing spondylitis disease activity index, *PASI* Psoriasis area and severity index, *FRAX Major* 10-year probability of a major osteoporotic fracture as assessed by the FRAX tool, *FRAX Hip* 10-year probability of a hip osteoporotic fracture as assessed by the FRAX tool, *BMD* Bone mineral density, *CTx* C-terminal telopeptides of type-I collagen, *PINP* Procollagen type I amino-terminal propeptide, *PTH* Parathyroid hormone, *25OHD* 25-hydroxyvitamin D. *Femoral neck BMD assessment was not performed for one patient as she had bilateral total hip replacement

**Table 2:** T-score categories based on femoral neck and lumbar spine areal bone mineral density (BMD), and distal radius volumetric BMD in PsA patients

Region of Interest	Normal (n,%)	Low Bone Mineral Density (n,%)	Osteoporosis (n,%)
Femoral neck (*n* = 117)	77 (66%)	35 (30%)	5 (4%)
L1-L4 lumbar spine	82 (70%)	32 (27%)	4 (3%)
Distal Radius (total)	14 (12%)	66 (56%)	38 (32%)

Normal: T-score ≥ −0.99; Low Bone Mineral Density: T-score between −1.00 and − 2.49; Osteoporosis: T-score ≤ −2.50

**Table 3:** Correlation analysis between areal and volumetric bone mineral density

			Areal			Volumetric Distal Radius	
	Region of Interest		Femoral Neck	L1-L4	Total	Trabecular	Cortical
**Areal**	**Femoral neck**	Spearman’s ρ	1.000	0.526	0.496	0.374	0.422
		*p* value	–	< 0.001	< 0.001	< 0.001	< 0.001
	**L1-L4**	Spearman’s ρ	0.526		0.488	0.343	0.421
		*p* value	< 0.001		< 0.001	< 0.001	< 0.001
**Volumetric Distal Radius**	**Distal Radius (total)**	Spearman’s ρ	0.496	0.488		0.601	0.842
		*p* value	< 0.001	< 0.001		< 0.001	< 0.001
	**Trabecular**	Spearman’s ρ	0.374	0.343	0.601		0.377
		*p* value	< 0.001	< 0.001	< 0.001		< 0.001
	**Cortical**	Spearman’s ρ	0.422	0.421	0.842	0.377	
		*p* value	< 0.001	< 0.001	< 0.001	< 0.001	

Fracture risk characteristics used in the FRAX tool are presented in Table 4 for PsA patients (*n* = 100) between 40 and 90 years of age. A 10 year probability of ≥20% for major osteoporotic fracture and 10 year probability of ≥3% for hip fracture was observed in 1 (1%) and 8 (8%) patients, respectively. One patient had probability above treatment threshold for both fracture types.

**Table 4:** Patients’ fracture risk characteristics used in the FRAX tool

Risk Factors	Patients between 40 and 90 years of age (***n*** = 100)
Age, years (median, range)	57 (40–85)
Male:Female	42:58
Weight (kg) (median, range)	83 (48–125)
Height (cm) (median, range)	164.5 (150–188)
Previous Fracture (n, %)	22 (22%)
Parent Fractured Hip (n, %)	4 (4%)
Current Smoking (n, %)	10 (10%)
Glucocorticoids (n, %)	20 (20%)
Rheumatoid Arthritis (n, %)	0 (0%)
Secondary Osteoporosis (n, %)	16 (16%)
Alcohol 3 or more units/day (n, %)	0 (0%)
Femoral neck areal BMD, g/cm^2^ (median, range) (*n* = 99)	0.934 (0.607–1.251)
FRAX Major, % (median, range)	3.75 (0.7–32)
FRAX Hip, % (median, range)	0.4 (0–16)
10 year probability of major osteoporotic fracture ≥20% (n, %)	1 (1%)
10 year probability of hip fracture ≥3% (n, %)	8 (8%)

A total of 53 (44.9%) and 62 (52.5%) of the patients were on conventional (Methotrexate, Leflunomide, Hydroxychloroquine or Sulphosalazine) and biologic (Infliximab, Adalimumab, Etanercept, Rituximab, Abatacept, Tocilizumab, Certolizumab, Golimumab or Ustekinumab) disease-modifying anti-rheumatic drugs (DMARD), respectively, either as monotherapy or in a combination. Three (2.5%) PsA patients did not receive therapy as they were suffering from malignant conditions (prostate cancer, breast cancer and malignant melanoma).

Upon univariate analysis of the PsA cohort data, patients with lower FN areal BMD were older, with longer duration of psoriasis and arthritis disease duration, and those with higher FN areal BMD had higher body mass index (BMI) (*p* < 0.05); women had significantly lower L1-L4 areal BMD (*p* < 0.05); those with lower DR total volumetric BMD were older and had longer menopause duration (*p* < 0.05); older patients and women had lower DR trabecular volumetric BMD and those with higher DR trabecular volumetric BMD had longer fertility duration (*p* < 0.05); those with lower DR cortical volumetric BMD were older (*p* < 0.05); the 10 year probability of major osteoporotic fracture was higher in patients with more severe disease as evaluated by DAS28, longer psoriasis and arthritis disease duration, and menopause duration (*p* < 0.05); and the 10 year probability of hip fracture was higher in patients with longer psoriasis and arthritis disease, and menopause duration, in those on conventional DMARDs and insufficient vitamin D status with 25OHD levels < 75 nmol/L or < 50 nmol/L (*p* < 0.05) (Table 5).

**Table 5:** Comparison of PsA patient subsets by univariable analyses

Dependent variable	Independent variable	Univariate analysis		
		***B*** (95% CI)	***β***	***p*** value
Femoral neck areal BMD	Age	−0.003 (− 0.005 - -0.001)	−0.307	0.001
	Psoriasis duration	−0.002 (− 0.005–0.000)	− 0.211	< 0.001
	Body Mass Index	0.004 (0.000–0.009)	0.180	0.050
	Arthritis duration	−0.005 (− 0.008 – − 0.002)	−0.286	0.002
L1-L4 areal BMD	Sex (women vs. men)	−0.068 (− 0.139 - -0.001)	−1.174	0.049
Distal radius total volumetric BMD	Age	−1.563 (−2.397 - -0.729)	−0.326	< 0.001
	Menopause duration	−2.213 (−4.070 - -0.356)	− 0.352	0.021
Distal radius trabecular volumetric BMD	Age	−0.836 (−1.491 - -0.181)	− 0.228	0.031
	Sex (women vs. men)	−24.338 (−41.172 - -7.505)	− 0.257	0.005
	Fertility duration	2482 (0.124–4.480)	0.315	0.040
Distal radius cortical volumetric BMD	Age	−2.116 (−3.376 - -0.857)	−0.295	0.001
10 year probability of major osteoporotic fracture	DAS28	1.351 (0.379–2.323)	0.277	0.007
	Psoriasis duration	0.085 (0.001–0.170)	0.198	0.048
	Arthritis duration	0.165 (0.045–0.286)	0.265	0.008
	Menopause duration	0.348 (0.142–0.554)	0.470	0.001
	cDMARD vs bDMARD	−2.890 (−5.049 - -0.731)	−0.259	0.009
10 year probability of hip fracture	Psoriasis duration	0.054 (0.008–0.100)	0.230	0.021
	Arthritis duration	0.080 (0.013–0.146)	0.233	0.020
	Menopause duration	0.178 (0.051–0.305)	0.405	0.007
	cDMARD vs bDMARD	−1.664 (−2.841 - -0.487)	−0.273	0.006
	< 75 nmol/L vs ≥75 nmol/L 25OHD	−1.800 (−3.317 - -0.284)	− 0.232	0.020
	< 50 nmol/L vs ≥50 nmol/L 25OHD	− 1.405 (− 2.598 - -0.213)	−0.230	0.021

*BMD* Bone mineral density, *DAS28* Disease activity Score in 28 joints, *cDMARD* Conventional disease-modifying anti-rheumatic drugs, *bDMARD* Biologic disease-modifying anti-rheumatic drugs, *25OHD* 25-hydroxyvitamin D

Multiple linear regression analyses revealed that age was an independent predictor of both areal and volumetric BMD. Additionally, BMI and arthritis disease duration also predicted FN areal BMD, female sex was an independent predictor of DR trabecular volumetric BMD. Disease activity score (DAS28) predicted the 10 year probability of major osteoporotic fracture and conventional DMARD predicted the 10 year probability of hip fracture (Table 6).

**Table 6:** Multiple regression analysis of bone mineral density and 10-year fracture probability

Dependent variable	Independent variable	Multivariable analysis		
		***B*** (95% CI)	***β***	***p*** value
Femoral neck areal BMD	Age	−0.004 (− 0.006 - -0.002)	−0.334	< 0.001
	Body Mass Index	0.006 (0.002–0.011)	0.266	0.003
	Arthritis duration	−0.003 (− 0.006–0.000)	−0.184	0.039
Distal radius total volumetric BMD	Age	−1.563 (−2.397 - -0.729)	−0.326	< 0.001
Distal radius trabecular volumetric BMD	Age	−0.905 (−1.538 - -0.272)	− 0.247	0.005
	Sex (women vs. men)	−25.947 (−42.333 - -9.560)	−0.274	0.002
Distal radius cortical volumetric BMD	Age	−2.116 (−3.376 - -0.857)	−0.295	0.001
10 year probability of major osteoporotic fracture	DAS28	1.351 (0.379–2.323)	0.277	0.007
10 year probability of hip fracture	cDMARD vs. bDMARD	−1-139 (−2.270 - -0.009)	−0.187	0.048

*BMD* Bone mineral density, *DAS28* Disease activity Score in 28 joints, *cDMARD* Conventional disease-modifying anti-rheumatic drugs, *bDMARD* Biologic disease-modifying anti-rheumatic drugs

The prevalence of hypovitaminosis D (25OHD < 75 nmol/L) was 79 and 58% in the PsA and control groups, respectively. A significant association was found between hypovitaminosis D and PsA; the odds for PsA patients to suffer with hypovitaminosis D was 2.74 (95%CI: 1.54–4.85, *p* < 0.001).

## Discussion

Both areal and volumetric bone mineral density in our patient population was significantly lower than the age and sex-matched controls. This finding is in agreement with a number of studies that have reported PsA patients with an increased risk of low areal bone mineral density [23–28]. But simultaneously is in disagreement with another series of studies that did not report low areal BMD [29–34]. This dichotomy may be due to the non-consistent comparison groups and reported outcomes. Our finding of FN areal BMD significantly correlating with disease duration supports similar previous findings [25, 32, 35].

Bone mineral density measured by pQCT have been reported previously by Kocijan et al. [36]. Kocijan et al. reported that trabecular and not cortical density was significantly lower in the patient population as compared to the controls, this finding is in contrast to our results of decreased trabecular and cortical density in the patient population. A probable explanation for this discrepancy may be due to the fact that our patient cohort is older, with longer psoriasis and arthritis disease duration. The present study is the first where areal BMD has been compared to volumetric BMD in PsA patients, with statistically significant correlation between the two methodologies. This finding is in tally with 2 other studies in patients with inflammatory rheumatic disease [14, 15].

We observed a significantly increased 10-year probability of both major and hip osteoporotic fractures as assessed by the FRAX tool in the studied cohort with psoriatic arthritis. Probability of fragility fractures has not been reported previously in PsA patients using the FRAX tool. Our probability findings are in concordance with findings where osteoporotic fractures were studied as primary endpoints, reporting higher odds of diagnosis with pathological fractures and elevated risk of all fractures [23, 37, 38]. A cross-sectional study from Spain reported increased prevalence of fragility fractures in postmenopausal PsA patients [29]. A Brazilian study reported longer disease duration as predictor of low-impact fractures [27]. Nonetheless, an Italian study reported no difference in the prevalence of fragility fractures between cases and controls [39].

Beside known predictors of the 10-year probability of fragility fractures, i.e., age and BMD, our findings suggest that in PsA, severe disease activity as assessed by DAS28 is also a noteworthy risk factor.

Fracture risk assessment using the FRAX tool and DR volumetric BMD measurement are excellent alternatives when FN BMD cannot be measured, as in our study where one patient had total bilateral hip replacement.

Although patients identified as being osteoporotic with FN areal BMD measurement were also classified as osteoporotic with DR volumetric BMD measurement, volumetric measurements identified a significantly greater number of patients with low bone mineral density (34% vs. 88%, *p* < 0.001). Although manufacturer provided German reference population is used to derive the T-score with both methodologies, the absence of agreement has also been reported by Marshall et al. [40].

Fracture risk assessment using the FRAX tool identified more patients deserving anti-osteoporosis treatment as compared to FN areal BMD assessment (*n* = 8 vs. *n* = 6). Patients with major osteoporotic or hip fracture probability in the intervention range, i.e., ≥20% (*n* = 8) and ≥ 3% (*n* = 1), respectively, were also osteoporotic when assessed for DR volumetric BMD, nonetheless, a wide discrepancy was noticed as a significant proportion of the cohort with non-intervention level FRAX probability was identified as osteoporotic (*n* = 28, 24%). The FRAX tool offers optional inclusion of FN areal BMD, and its clinical utility in identification of those at increased risk of fragility fractures may be improved were volumetric BMD values and psoriatic arthritis, as a secondary risk factor, also facilitated in the calculation of fracture probability.

The FRAX tool is designed to assess those between 40 and 90 years of age, given this inherent limitation the fracture probability of the young cannot be assessed. Among those under 40 years of age (*n* = 18), areal FN BMD assessment identified 3 (17%) and DR volumetric BMD examination identified 16 (89%) psoriatic arthritis patient with low bone density (T-score ≤ − 1.0). Our observation suggests that volumetric BMD assessment better identifies those at increased fracture probability, and offers opportunity to initiate fracture risk reduction intervention promptly at a younger age. The true burden to osteoporosis may be underestimated with areal BMD measurement alone.

Although beyond the scope of this present study, it may be worthwhile to examine the utility of diagnostic imaging techniques such as ultrasonography of the knee and magnetic resonance imaging in PsA disease severity assessment [41–44].

The Hungarian National Healthcare System subsidises antiosteoporotic therapy for those with an osteoporotic T-score, based on the WHO classification (T-score ≤ − 2.5), or with FRAX probability of more than 3 and 20% for hip and major osteoporotic fracture, respectively. Our results suggest that a number of osteoporotic and osteopenic patients deserving fracture risk reducing intervention are missed using areal BMD measurement alone, and as such FRAX assessment.

As compared to the control groups, the studied biochemical markers of bone turnover were significantly elevated suggesting a high bone turnover in the PsA population. This finding is supported by one previous study [45]. Grisar et al. reported that CTx levels were significantly higher in the PsA group as compared to the healthy controls [30]. Szentpetery et al. reported correlation between the studied bone markers and hand BMD [46]. Borman et al. reported correlation between CTx and duration of arthritis and no difference in marker levels comparing patients with and without arthritis [28]. Nonetheless, in our study we found no correlation between the studied parameters and bone markers. The inconsistency in bone marker results in the numerous studies published has been summarized in a review by Jadon et al. [47].

The finding of high prevalence of hypovitaminosis D in our study cohort is in agreement with previous studies [45, 48–50]. Nonetheless, no difference in 25OHD levels was reported upon comparison of psoriasis patients with and without arthritis in one previous study and another study found lack of correlation between 25OHD levels and the disease activity score [45, 49]. A predisposition for hypovitaminosis D in the PsA cohort may be due to the debilitating nature of the condition, henceforth, they may involve only in limited physical activity; furthermore, patients may shy away from outdoor activity given the mental burden of the skin manifestations of the disease.

Although not supported by correlation analysis in our study cohort, hypovitaminosis D, high bone turnover and low bone mineral density may contribute to the increased fragility fracture probability in this population.

Our study has limitations. Given the difficulties in getting access to the local population register and absence of commercially available population registers, our control group recruitment method may not have been unbiased. Validation of our results is deemed mandatory optimally with a substantially larger cohort.

A higher number of study participants could have improved the statistical power of our analyses, nonetheless, we report a high 10-year probability of fragility fractures along with an increased prevalence of hypovitaminosis D in a PsA cohort with low bone mineral density and high bone turnover; additionally, the case-control study design minimizes the effect of confounding risk factors.

Although warranting validation, the clinical utility of volumetric BMD examination complemented with traditional DXA-based areal BMD measurement and FRAX assessment, are readily applicable in the PsA patient population and serve as an inexpensive tool in identifying patients with low bone mineral density. Prompt identification, treatment and follow-up of patients at risk would help in reducing the burden of fragility fractures in the PsA patient population.

## Conclusion

In the studied PsA cohort, disease activity was an independent predictor of 10-year probability for a major osteoporotic fracture, and complemented assessment of volumetric and areal BMD assured better efficacy at identifying those with low bone mineral density.

## Data Availability

The datasets used and/or analysed during the current study are available from the corresponding author on reasonable request.

## Abbreviations

25OHD: 25-hydroxyvitamin D
BASDAI: Bath ankylosing spondylitis disease activity index
bDMARD: Biologic disease-modifying anti-rheumatic drugs
BMD: Bone mineral density
BMI: Body mass index
CASPAR: Classification criteria for psoriatic arthritis
cDMARD: Conventional disease-modifying anti-rheumatic drugs
CLIA: Chemiluminescence immunoassay
CTx: C-terminal telopeptides of type-I collagen
CV: Coefficient of variation
DAS28: Disease activity score in 28 joints
DXA: Dual energy X-ray absorptiometry
DMARD: Disease-modifying anti-rheumatic drugs
DR: Distal radius
ESR: Erythrocyte sedimentation rate
FN: Femoral neck
FRAX: Fracture risk assessment
L1-L4: Lumbar vertebrae L1 to L4
OC: Osteocalcin
OTKA: Hungarian national scientific research fund
PASI: Psoriasis area and severity index
PINP: Procollagen type I amino-terminal propeptide
pQCT: Peripheral quantitative computed tomography
PsA: Psoriatic arthritis
PTH: Parathyroid hormone
WHO: World Health Organization
